# Frank J. Wethered

**Published:** 1928

**Authors:** 


### Obituary.

---

### FRANK J. WETHERED, M.D. (Lond. and Bristol), F.R.C.P.

Though all his active professional work was as a consulting physician in London, the death of Frank Wethered, at the age of 68, recalls happy memories to many of his old fellow-students at the Bristol Medical School, before he went on to complete his student career at the London Hospital, and at Berlin and Vienna.

During the earlier days at Bristol he was ever careful and thorough in his work. He was a keen amateur actor, and took a very active share in initiating the Bristol Medical Dramatic Society with a two-night performance of "London Assurance" at the Alexandra Hall, when over £500 was collected for our medical charities.

In these early days he was especially interested in histology, and shortly after his appointment as Medical Registrar and Demonstrator of Practical Medicine at Middlesex Hospital in 1892 he published a text-book on *Medical Microscopy.*

In 1899 he was appointed Assistant Physician, and in 1913 Physician to this Hospital, and he was furthermore appointed a member of the staffs at Brompton Hospital for Consumption and St. Saviour's Hospital.

His work, especially at Brompton, brought him constantly in contact with the late Sir Richard Douglas Powell, with whom he became more closely associated in private practice. His knowledge of pulmonary tuberculosis led him to compete for the King's Competition with an essay on "A Sanatorium for Consumption," for which he was awarded the second prize.

Only the older practitioners can recall the furore of hope aroused by the announcement of Professor Koch's claims for his tuberculin, but Sir Douglas Powell was the one English physician to be accorded a small amount to bring back from his visit to Berlin. As Frank Wethered was then acting as his assistant he was charged with the carrying out of the method, and so the writer was enabled to organise a demonstration of the technique by Frank Wethered at the Bristol Royal Infirmary, Sir Douglas Powell promising sufficient tuberculin for the continued treatment of two patients. Our Board Room was crowded out by the practitioners who flocked to Dr. Wethered's demonstration, a sure indication of the intense and widespread interest that had been aroused.

His next association with Bristol arose many years later, when in 1913 he obtained the M.D. Bristol.

During the war he threw his energies into his work as Captain, R.A.M.C. (T.F.), in the Third London General Hospital.

In 1889 he married Miss How, and had one daughter, to whom he was devoted. The sudden and tragic death of his only child in 1918 was a terrible grief, and shortly after he resigned all his appointments in London and retired to Falmouth, where he became Consulting Physician to the Royal Cornwall Infirmary and undertook some consulting work.

For a few years past his health had been failing, and he suffered from anginal attacks. He will be remembered by a large circle of friends for his kindly, genial disposition and charming personality.

